# Head position control strategies in progressive Supranuclear Palsy versus Idiopathic Parkinson’s Disease during dynamic-on-static platform tilt

**DOI:** 10.3389/fneur.2024.1477493

**Published:** 2025-04-16

**Authors:** Stefan Kammermeier, Kathrin Maierbeck, Lucia Dietrich, Annika Eissner, Stefan Lorenzl, Arun Singh, Kai Bötzel, Christoph Maurer

**Affiliations:** ^1^Klinikum der Universität München, Ludwig-Maximilians-Universität, Neurologische Klinik und Poliklinik, München, Germany; ^2^Klinikum der Universität München, Klinik für Anästhesiologie, München, Germany; ^3^Abteilung für Allgemeinchirurgie, Kliniken Ostallgäu-Kaufbeuren, Kaufbeuren, Germany; ^4^Abteilung für Neurologie, Krankenhaus Agatharied, Hausham, Germany; ^5^Division of Basic Biomedical Sciences, Sanford School of Medicine, University of South Dakota, Vermillion, SD, United States; ^6^Klinik für Neurologie und Neurophysiologie, Universitätsklinikum Freiburg, Freiburg im Breisgau, Germany

**Keywords:** Idiopathic Parkinson’s Disease, Progressive Supranuclear Palsy, falling, posturography, head stabilization

## Abstract

**Objectives:**

We investigated differences in head stabilization among Progressive Supranuclear Palsy (PSP), advanced Idiopathic Parkinson’s Disease (IPD) and healthy controls during passive anteroposterior platform tilting to determine factors for disease-specific falling.

**Methods:**

Seventeen PSP, eleven IPD and eighteen control subjects were exposed to pseudorandom multi-frequency antero-posterior platform tilts, while recording 3D motion of body segments with a Zebris ultrasound positioning system. Fourier transforms were computed from the time series datasets to assess transfer functions between stimuli (platform tilts) and responses (angles of the head, trunk and hip in space).

**Results:**

Overall head excursions in space among PSP was several times increased in relation to IPD and controls. The stimulus driven contribution to the head movement, i.e., the GAIN of the transfer function between platform stimulus and head movement, was double the amount of the values derived from IPD and 5-fold relative to controls. GAIN of the transfer function was the highest among the middle tilt frequencies 0.15–0.4 Hz, and was independent from the eyes open vs. closed condition.

**Conclusion:**

PSP patients’ head excursions with respect to the shoulder girdle and trunk were exceptionally increased, compared to IPD and controls. The source for the larger excursions, however, was not related to an unspecific lack of head stabilization, but was instead determined by a central strategy. Consistent with pathoanatomical degeneration of mesencephalic supracollicular pathways processing visual flow or vestibular pathways, PSP resorted to egocentric proprioceptive-dominated stabilization to the surface, rather than allocentric stabilization in space like IPD. Passive neck rigidity in PSP did not contribute significantly.

**Significance:**

The axial muscle rigidity emphasized in PSP versus IPD did not contribute to body instability in the sensory context of unstable surfaces. Instead, deficits in processing of visual and vestibular information played a larger role in PSP falling than previously known.

## Introduction

Progressive Supranuclear Palsy (PSP) and Idiopathic Parkinson’s Disease (IPD) are frequent neurodegenerative disabling movement disorders with postural instability, both with significant negative impact from falls during different stages of their disease progression.

Idiopathic Parkinson’s Disease (IPD) is an alpha-synucleinopathy resulting in a hypokinetic rigid motor syndrome (1–4) with initially well-treatable hypokinesia, rigidity and tremor. Usually after 5–8 years, postural control is increasingly affected with motor freezing. Falling at advanced stages occurs mostly forward, concurring with stooped posture in generalized flexion and freezing phenomena. This is likely associated with degeneration of non-dopaminergic structures (1, 5, 6). Falling in early stages has also been described, and provoked falls in any direction may occur at all disease stages (1, 7–9). Additional orthostatic dysfunction (10) and frontal executive disorders (1, 11) increasingly exhibited along the course of the disease contribute to postural deficits.

The most frequent atypical Parkinsonism syndrome (PSP) is a tauopathy affecting particularly the midbrain area, resulting in a clinically typical supranuclear vertical gaze disorder, along with impairment of supracollicular and vestibulospinal pathways (8, 12–17). All symptoms, including postural instability, respond poorly to dopaminergic medication, and frequent falls occur within the first disease year. Falls are typically unprovoked, backwards and without reflexive countermeasures, injuring mostly the back of the head with considerably higher morbidity and even mortality, compared to IPD (18, 19). PSP patients report they cannot foresee these sudden falls, whereas IPD patients become fully aware that postural limits are exceeded, and a fall is imminent (16, 18–20). Aspects of dysautonomia contribute additionally (21). Aforementioned typical features describe the most frequent subtype (about 60%, referred to as Richardson’s Syndrome PSP-RS); further clinical subtypes with, e.g., initial Levodopa-therapy response as PSP-Parkinsonism (PPP-P) have been established (13, 22) and pose a particular diagnostic challenge in the differentiation to early stages of IPD due to an initially adequate Levodopa response, which is lost during disease progression. The purview of this study remains focused on the most frequent subtype PSP-RS and its postural deficits.

Stable upright stance relies on multisensory integration (23–27) of the vestibular (angular acceleration, directional velocity and gravity-related tilt) and the visual system positioned in the head (vertical and horizon alignment; optic flow), in conjunction with the joint and muscle tissue proprioceptors positioned throughout the body. Higher-order intrinsic and head-referenced coordinate systems of the body in space and vertical alignment surpass the bandwidth and resolution limitations in both the temporal and amplitude domain of the respective sensory modalities [for studies on multisensory integration and sensory capability weighting, see e.g., (24, 27–53)]. Especially the wide mobility range of the head with its contained sensors relative to the body requires re-alignment of spatial coordinate systems of the head-based sensors to a body-centered coordinate system, particularly relying on neck proprioceptive inputs (54). Previous findings point to brainstem and cerebellar midline structures as core elements of this mechanism (29). One goal of postural control is to maintain the position of the head with a minimum of absolute motion in space, with the objective of an optimized sensory acquisition range through low temporal-resolution sensors [visual optic flow and vestibular otolith afferents; (27–29, 46)].

Recent studies have focused on the pathophysiological postural differences between still ambulatory PSP with backward falling versus advanced stage IPD with mostly forward-directed falling, since both conditions correspond to the respective last stages of disease prior to permanent immobilization and eventual immobility-related, frequently infectious causes of morbidity and mortality. Their objective was to define quantifiable deficits in relatively simple device-based examinations, which could serve as quantitative effect markers for possible emergent therapeutic interventions aiming to extend the disease stage of basal mobility just prior to permanent immobilization and its dire consequences when given during already manifest disease. Unless affordable, large population-based screening tools become available to determine individuals with future manifestation of neurodegenerative diseases in conjunction with primary prophylactic therapies to prevent their onset, prospective therapies in the near to intermediate future will likely target individuals with already manifest disease and a spectrum of relevant clinical deficits. Examinations among these individuals must balance safety concerns in posturally unstable patients with the necessary amplitudes of postural sensory challenges to provoke disease-specific response abnormalities. This was the objective of the previous studies conducted by our group [particularly (55–57)].

During passive anteroposterior 0.5 to 1° small-amplitude platform tilting at multiple frequency bands among the same patients investigated in the current study, individuals with PSP displayed pathologically altered response gain with particularly exaggerated swaying of the body above the hip at both high and low frequencies, and a stabilization strategy focused on the proprioceptive sensory input, which attempted to stabilize the body orthogonal to the tilting platform surface (56). Results by Liao and Ondo point toward altered vestibular otolith sensory processing in PSP (14, 15). Another study in PSP and IPD found the tonic neck reflexes required for adequate proprioceptive postural control during neck vibration surprisingly intact in PSP (55). Dale and colleagues (58) discovered specific reduced conscious sensitivity to passive body backward versus forward platform tilt in PSP; an effect which could not be found in IPD and healthy controls. Additional deficits appear to influence motor reafference control, since the same PSP patients investigated in this study appeared to respond with exaggerated high-frequency postural efforts to self-triggered arm-raising with small weights <2% of body mass by Kammermeier et al. (57).

IPD patients by contrast pursued a vestibular-centered approach to stabilize the body vertically in space against the moving platform (56), in alignment with studies indicating that individuals with IPD preferred visual and vestibular postural guidance (4, 25, 59–61).

In the study presented here, we investigated three-dimensional head motion in patients suffering from advanced-stage IPD and ambulatory early PSP during small-amplitude and multi-frequency tilts of the body support surface, to differentiate head stabilization deficits between these disorders. Whether the motion of the head in these diseases followed an own strategy for body stabilization, and if these motions constituted an attempt to ameliorate certain disease-based sensory processing deficits, or rather occurred as a part of sensory processing deficits by themselves, were subject of this study’s purview.

## Materials and methods

### Subjects

**Figure 1:**
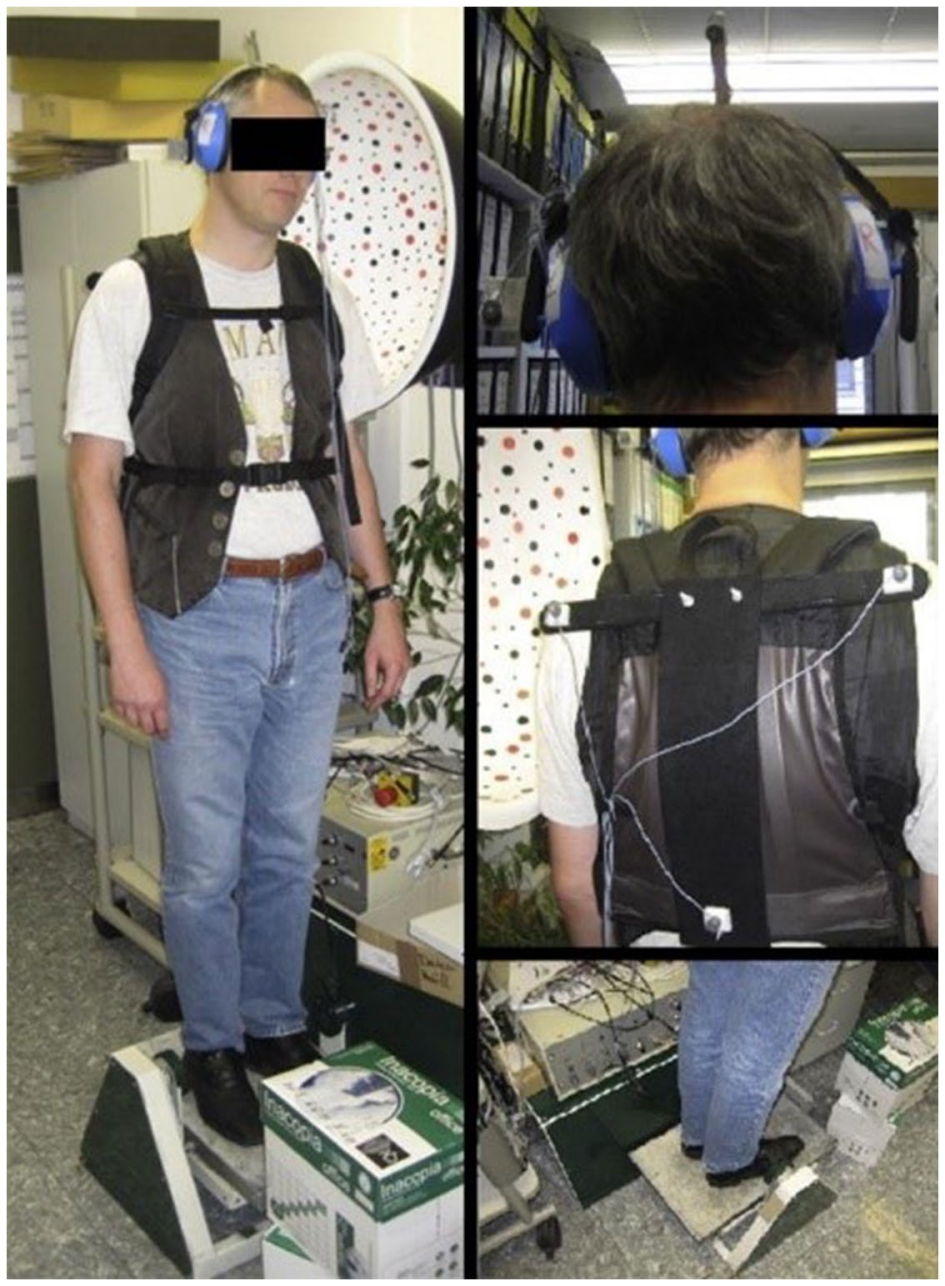
The experimental setup. The test subject stands on a platform, tilting in the sagittal plane around the ankle. The subject’s feet were placed within marked positions, with the heels together and the tips spread 15° apart, while the arms hung loosely by the sides. Photography depicting a university employee unrelated to the study instead of a study subject in the experimental setup attire; figure previously displayed in Kammermeier et al. (56). Publication use approved by the consenting individual.

Three groups were recruited for a series of related studies; photographies of the experimental setup are depicted in Figure 1. Subject demographics and clinical scores are summarized in Table 1. All participants gave written informed consent and data was anonymized at study inclusion, in accordance with the Helsinki Declaration and the local ethics committee (decision No. 142/04; Ethikkommission der Medizinischen Fakultät). This study collective is identical with subjects presented in Kammermeier et al. (56).

**Table 1:** Idiopathic Parkinson’s Disease (IPD) and Progressive Supranuclear Palsy (PSP) subjects are shown with age, sex, height, weight, and where applies Unified Parkinson’s Disease Rating Scale UPDRS scores of category I, II, motor subscore III (UPDRS III in IPD: 19.3 ± 8.9, in PSP 14.8 ± 3.2, *p* = 0.07), modified III, and total UPDRS with modified subscore III, as well as individual subscores 3.3 (limb and neck rigidity), 3.9 (arising from chair), 3.10 (gait), 3.12 (postural stability); SEADL, PIGD, and Hoehn & Yahr (H&Y in IPD: 2.3 ± 0.6; in PSP 2.5 ± 0.3, *p* = 0.21); Progressive Supranuclear Rating Scale PSPRS, scale of the NNiPPS study (Neuroprotection and Natural History in Parkinson Plus Syndromes), Frontal assessment battery FAB, Mini-Mental State Examination MMSE and Montgomery-Åsberg depression rating scale MADRS.

	Age	Height (cm)	Weight (kg)	UPDRS1	UPDRS2	UPDRS3	UPDRS mod3	Total with modified 3	UPDRS 3.3 Rigor Extr	UPDRS 3.3 Rigor Neck	UPDRS 3.9 Arising	UPDRS 3.10 Gait	UPDRS 3.12 Stability	PIGD	Hoehn & Yahr	BBS	Golbe	PSP-staging	NNiPPs	FBA	MMSE	PSP-RS	SEADL	MADRS	Disease duration	Ldopa	Rasagiline verum
IPD5	66	177	77	3	14	18	12	29	1	0	0	2	0	5	2.5										4	1	
IPD6	72	170	72	2	21	19	10	33	1	0	0	3	0	6	3										15	1	
IPD7	72	176	81	3	15	34	20	38	3	3	2	1	1	9	2.5										9	1	
IPD8	73	157	65	1	16	24	14	31	1	0	0	2	0	4	2										12	1	
IPD9	71	176	93	1	23	15	11	35	1	0	1	2	1	6	1										14	1	
IPD11	66	180	75	2	9	9	7	18	1	0	0	1	0	3	2										10	1	
IPD13	69	168	69	2	12	7	7	21	1	0	0	1	0	3	1.5										6	1	
IPD15	63	179	66	2	20	14	13	35	0	0	0	3	3	12	3										18	1	
IPD17	72	167	48	2	14	19	12	28	1	0	0	2	0	6	2.5										15	1	
IPD18	70	181	84	2	13	35	18	33	2	0	0	0	0	2	2.5										8	1	
IPD20	66	162	62	0	10	18	13	23	1	0	0	1	0	6	3										10	1	
PSP1	68	174	76	6	10	14	14	30	0	2	1	0	2	7	2.5	34	34	2	34	14	29	34	50	13	5	1	0
PSP2	74	168	68	2	11	15	15	28	1	2	1	0	1	6	2.5	46	30	2	24	17	30	27	90	11	1	1	0
PSP3	70	172	87	0	13	19	15	28	2	1	0	0	1	7	2.5	49	22	2	23	14	29	26	80	10	24	1	1
PSP4	60	176	65	4	7	11	10	21	2	0	2	1	2	14	3	53	23	2	25	16	29	27	90	15	1	1	1
PSP6	70	156	56	2	14	13	26	42	1	2	1	0	1	12	3	47	24	2	36	13	26	29	70	13	2	1	0
PSP7	60	164	123	1	8	13	11	20	1	2	0	0	1	6	2.5	53	17	2		16	30	19	90	4	6	1	0
PSP8	74	165	56	1	7	14	13	21	1	1	1	1	3	12	2.5	49	17	2	17	18	29	18	90	6	5	0	0
PSP10	66	168	55	3	10	9	9	22	1	2	0	0	1	5	2.5	46	18	2	30	13	30	23	90	26	1	0	1
PSP12	65	162	62	6	12	17	16	34	0	2	1	0	1	6	2.5	45	27	2	34	13	28	30	70	24	4	1	0
PSP14	65	161	60	1	13	15	13	27	2	2	1	0	2	9	3	49	26	3	23	15	28	26	80	1	4	0	1
PSP15	65	162	70	2	17	14	14	33	0	1	1	0	2	7	2.5	54	35	2	26	18	30	31	70	15	6	1	1
PSP19	69	174	68	4	21	21	18	43	1	1	1	1	2	10	2.5	38	31	2	41	9	26	43	60	11	6	0	1
PSP21	70	178	82	2	12	13	5	19	1	0	0	0	2	3	2	41	24	2	34		28	38	70	5	6	1	0
PSP22	65	168	75	3	13	17	18	34	1	2	2	0	2	12	3	49	42	2	22	14	29	38	70	17	2	1	0
PSP23	64	162	68	2	16	13	13	31	0	2	2	1	1	9	2	42	34	2	26	14	30	39	80	12	22	1	0
PSP25	69	183	102	2	14	13	11	27	0	2	1	0	1	5	2	48	34	2	25	17	29	30	80	3	9	0	0
PSP26	69	167	52	2	15	20	20	37						12	3	43	36	2	24	15	26	35	70	9	3	1	1

Disease duration for IPD is given on a scale of years, for PSP disease duration since clinical onset is given in months (disease duration in IPD: 11.0 ± 4.2 y; in PSP 0.52 ± 0.55 y, *p* < 0.0001). All IPD received Levodopa (marked “1”); PSP subjects with a baseline Levodopa dosage are also indicated with “1” (otherwise “0”). PSP subjects in the PROSPERA study receiving Rasagiline verum are indicated with “1” in the corresponding section, those receiving placebo are marked “0.” Control subjects are not shown here.

17 Progressive Supranuclear Palsy patients of the Richardson type (PSP-RS) participated (67 ± 4.0 years old, ten female, seven male). All but one were also participants of the PROSPERA study (prematurely ended, randomized double-blinded Rasagiline in PSP, EudraCT number 2008-007520-26). All PSP patients were “Clinical Probable PSP” according to the NINDS-SPSP criteria valid at the time of study inclusion [(16); “Definite PSP” would require all criteria of “Clinical Probable” plus neurosurgical-bioptic or post-mortem histology]. Clinical testing included (additional to those tested in IPD): PSP Rating Scale PSPRS, the scale of the NNiPPS study (Neuroprotection and Natural History in Parkinson Plus Syndromes), Frontal assessment battery FAB, Mini-Mental State Examination MMSE and Montgomery-Åsberg depression rating scale MADRS. Most of these patients received a small baseline dose of Levodopa (indicated in Table 1) for limited fine motor improvement. Please note that in the meantime updated clinical criteria and further subtyping of PSP have been released by the Movement Disorder Society study group (13).

11 Idiopathic Parkinson’s Disease IPD patients participated (four female, seven male; 69 ± 3.3 years), with a 4+ years course of typical LDopa-responsive hypokinetic-rigid syndrome, and no clinical indications of atypical Parkinsonism, known postural instability in the pull test and falls more than once a month, in accordance with diagnostic criteria of the International Movement Disorder Society (62). Since IPD patients fall regularly even under their optimal medication, we investigated patients on their regular medication including Levodopa and dopamine agonists in relative ON, rather than creating an artificial OFF state that would not occur in daily life. Please note that IPD patients included here experienced their falls during both ON and relative OFF periods in daily life. None had deep brain stimulation. The momentary state of patients’ mobility was assessed just prior to the experiment with the Unified Parkinson’s Disease Rating Scale UPDRS, Hoehn & Yahr stage and the modified Schwab & England scale for recent capabilities in activities of daily living SEADL. Clinical examinations scores reflect this ON stage with respect particularly to hypokinetic-rigid symptoms. Rating and individual UPDRS items relevant to posture are noted in Table 1.

Healthy control subjects (CTR) were recruited from among family members of the patients, relatives of the authors and former university personnel. 19 participated (age 58 ± 9.1, 11 females and seven males). None had a history of neurological disorders of any sort, or orthopedic disorders requiring surgery or regular medication. Control subjects were significantly younger than IPD patients, and not significantly younger than PSP patients.

Due to publication constraints by PROSPERA, all patients were followed up for 4 years. None were re-diagnosed with a different Parkinsonism spectrum disorder; no control subject developed any form of neurological movement disorder.

### Dynamic posturography

All subjects stood on a Toennis dynamic tilt platform with integrated piezoelectric posturography element [designs out of production, support surface 38 × 58 cm, turning angle around the ankle joint, used, e.g., in (8, 56)]. This platform was used to disturb the proprioceptive information of both ankles by applying continuous support surface rotations around the ankle axis. The feet were placed together at the soles on the platform with the toes spread apart by 5 cm. Auditory exclusion was provided by earmuffs (Figure 1). The tilt stimulus was delivered from a personal computer running Matlab 14 (The MathWorks Inc., Natick, MA, USA).^1^ An anteroposterior (*y*-axis) center of foot pressure COP displacement signal at 100 Hz (surrogate parameter of center of mass COM) was received in turn.

### 3D motion analysis

**Figure 2:**
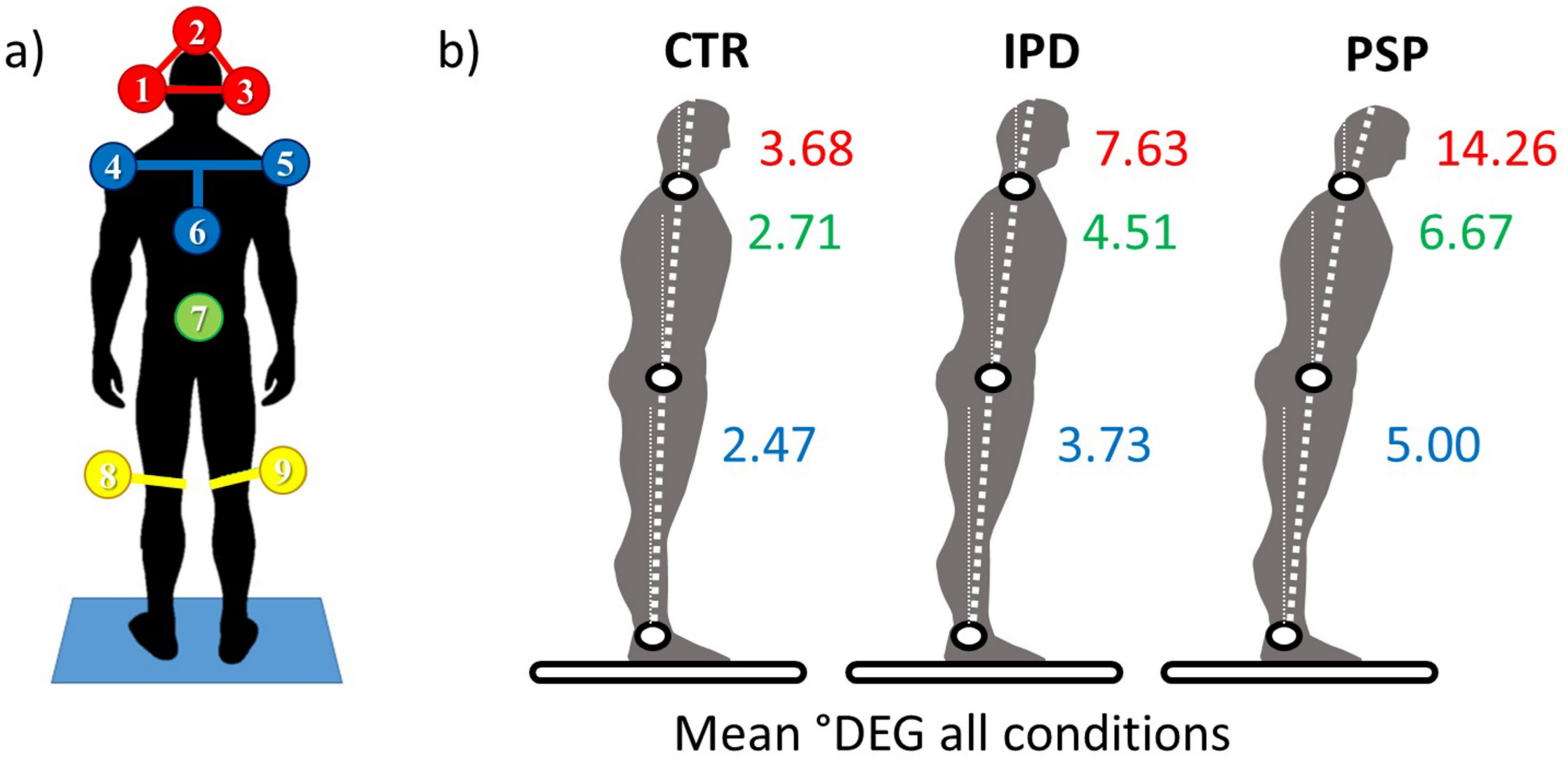
**(a)** 3D ultrasound position markers (Zebris system) placement viewed from behind in the perspective of the ultrasound receptacle. Head (1,2,3 in red), upper chest “trunk” (4,5,6 in blue), hip (7 in green), and lateral femoral epicondyles (8,9 in yellow). **(b)** Mean Amplitudes of angular excursions for aforementioned body segments among healthy controls CTR, IPD, and PSP; the statistical mean is derived from the entirety of datasets from angular displacement conditions (0.5°/1°) and for eyes open and eyes closed (EO/EC).

Zebris 3D real time ultrasound position markers were placed on the subjects, pointing backwards to define head (3 markers, one 5 cm over the vertex, the other two lateral to the ears positioned 4 cm away from the side of the skull and in line with the upper attachment point of the auricle), upper trunk (3 markers), hip (1 marker) and knee (1 marker 10 cm above each knee) motion and position during the experiments (placement see Figure 2a). The ultrasound receiver CMS20S was placed ~1 m behind subjects on the platform at roughly head level and subsequently micro-adjusted to get full coverage of all markers throughout the platform motion range (all products Zebris Medical GmbH, Isny im Allgäu, Germany).^2^ The innate Zebris software recorded the 3D position tracks concurrent with platform motion. Sampling rate was dynamic between 80 and 200 Hz, depending on momentarily detectable markers, and was resampled post-hoc to 100 Hz, the same as the platform pressure sensor acquisition frequency. Positions of left and right markers at head and shoulder level were combined by averaging to determine midsagittal positions.

Anthropometric data was collected for correlation with 3D marker positions and passive body mechanics representation in the subsequent analyses (height of the central marker above the head and both knee markers over the platform, body height and weight).

For this study, the position of the head element relative to the trunk element, each defined by their respective ultrasound markers, and the underlying platform were used as the primary variables.

### Broadband platform tilt

Platform rotations were designed as pseudorandom stimuli (PRTS, pseudorandom ternary sequence) with two peak angular displacements (0.5° and 1°, delivered in consecutive trials) presented at 11 frequencies (0.05, 0.15, 0.3, 0.4, 0.55, 0.7, 0.9, 1.1, 1.35, 1.75, and 2.2 Hz), which hamper habituation effects and reflect a frequency range to which the human body can adequately produce postural compensation reactions (56). The platform rotations were based on an 80 state pseudorandom ternary sequence of numbers with a time increment of 0.25 s. This sequence resulted in an angular velocity signal with a duration of 20 s [see e.g., (63)]. Integration of this signal resulted in an angular position signal that was used as the disturbance signal of the platform. The disturbance was repeated three times resulting in trials with a duration of 60 s. To eliminate transient effects, the first disturbance cycle of 20 s was removed from each trial, resulting in two cycles per trial. As each condition was performed twice, the signals of the disturbance command and the segment angles were segmented into four data blocks of 20 s [see (63)].

### Course of experiments

For each subject an eyes open (EO) and eyes closed (EC) trial were completed at 0.5° maximum angular displacement, followed by the next higher amplitude set at 1°. Note that these miniscule amplitudes were well within the stability margins of all subjects tested, even of the most severely affected PSP patients enrolled here. Stimuli were barely noticeable by the subjects. This related to PSP patients’ typical anamnestic statements, suggesting that falls were related to miniscule floor unevenness or even “no apparent reason at all.”

During each recording instance, 30 s of spontaneous undisturbed stance were followed without announcement by 60 s of platform tilting, and another 30 s of undisturbed stance. Each angular displacement (0.5°/1°) combined with each of the eye conditions (EO/EC) was performed twice.

### Data analysis

Data analysis was performed off-line with custom-made software programmed in MATLAB^®^ (The Math-Works Inc., Natick, MA, USA). For each condition, the responses of the head and trunk segments to the disturbance were obtained by estimating the sensitivity functions. To this end, the data blocks of the disturbance command signal and the segment angles were transformed to the frequency domain using discrete Fourier transform. Subsequently, the frequency coefficients were averaged across data blocks, yielding a mean disturbance signal and mean response signals in the frequency domain. The power spectral density of the disturbance signal as well as the cross-spectral density between the disturbance signal and the segment angles were calculated for the stimulated frequencies. For each condition and each subject, the sensitivity functions were estimated using the indirect approach by dividing the cross spectral density by the power spectral density. The resulting sensitivity function provides the Gain and Phase, which describe the response of the balance control system at each excited frequency. The Gain indicates how much the disturbance signal is amplified in the respective segment angle response signal, whereas the Phase indicates the phase lag of the responses relative to the disturbance signal. Furthermore, we calculated Coherence, a measure of reproducibility of the response. Technically, Coherence is calculated as the quotient between the cross-power spectrum of stimulus and response, and the product of the individual spectra of stimulus and response. Whereas a Coherence value of 0 indicates that there is no linear correlation between the stimulus and response, a Coherence value of 1 indicates a perfect linear correlation with no noise. Values less than 1 occur in practice either because there is noise in the system, or there is a nonlinear relation between stimulus and response.

The transfer functions used to describe body segment responses relative to multi-frequency platform motion are detailed in (64) (formulae 2–7; the respective portion is quoted in the Supplementary).

### Statistics

Statistics were performed using Matlab-generated output files from pre-processing into Microsoft Excel spreadsheets and subsequent analysis by a designated statistics application (JMP^®^ by SAS Institute Inc., Cary, NC, USA). Statistical significance was tested by a two-level analysis of variance (ANOVA), unless stated otherwise. The between-subjects factor was group (PSP, IPD and controls CTR), the within-subject factors were visual condition, stimulus amplitude, stimulus frequency, and body segment (head, shoulder, hip; each in relation to space). The level of statistical significance was set at alpha error of *p* = 0.05. Bonferroni corrections were applied for multiple comparisons.

## Results

Features of differential head mobility in PSP, IPD and controls are detailed with respect to relative mobility of the head segment across the frequency spectrum, augmentation and attenuation of the imposed stimuli by GAIN and latencies and anticipation of response in PHASE. The range of body segment motion for the respective conditions 0.5° and 1° platform tilt, each in eyes open and closed conditions (EO/EC) are depicted in Figure 2b.

### GAIN results

The responses of the head element to the tilt of the body with multiple superimposed frequencies can be described by the GAIN factor, defined as the amplification (positive value) or attenuation (negative value) of directional shift amplitude at a given frequency and PHASE lag.

**Figure 3:**
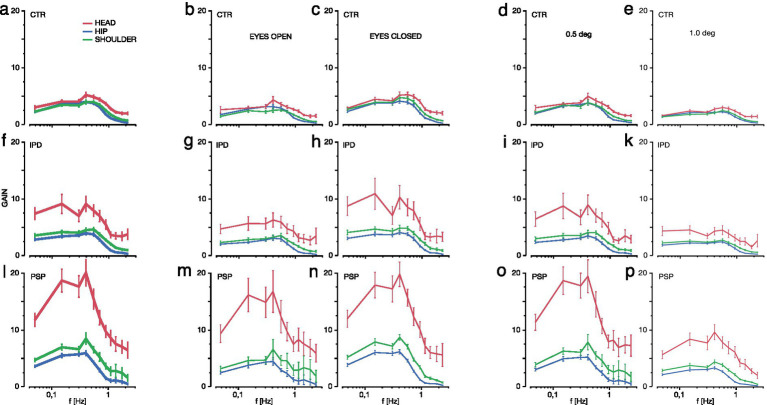
The GAIN factor of hip movements (blue), shoulder (green) and head (red, in ascending order by magnitude within each plot) is displayed for the range of platform frequencies contained in the multi-frequency tilt stimulus (on decalogarithmic scale) among control subjects (CTR, **a–e**, top line), Idiopathic Parkinson’s Syndrome (IPD, **f–k**, middle line) and Progressive Supranuclear Palsy (PSP, **l–p**, bottom line). Vertical whiskers indicate 95% confidence intervals. First column indicates GAIN for all conditions pooled **(a,f,l)**, second column for eyes open EO condition **(b,g,m)**, third column shows the eyes closed EC condition **(c,h,n)**, fourth column depicts all EO/EC conditions at 0.5° maximum platform tilt **(d,i,o)**, fifth and last column shows EO/EC pooled at 1° maximum platform tilt **(e,k,p)**.

The postural body response to angular displacement of the support was characterized by increasing displacement amplitudes along the multisegmental body model, as displayed in Figure 3 across all subject groups. GAIN was highest for the head, significantly higher than in the shoulder and hip across all groups and frequencies (*F* = 403.7, *p* < 0.0001, Figures 3a,f,l). Across all segments and frequencies, GAIN was the largest in PSP, smaller in IPD, and the smallest in controls (*F* = 273.5, *p* < 0.0001). Subjects group (PSP, IPD, CTR) and segment (Head, Shoulder, Hip) significantly interacted (*F* = 119.4, *p* < 0.0001). For example, Head GAIN was increased by a factor 2–3x in IPD and up to 4–5x in PSP, each relative to controls. Moreover, subject groups (PSP, IPD, CTR) significantly interacted with frequencies (*F* = 7.36, *p* < 0.0001), which was mainly caused by a relatively higher GAIN increase in frequencies below 0.7 Hz in PSP. Notably, PSP head GAIN factor was elevated up to a factor of 25 among the 0.15–0.4 Hz frequencies.

Overall, the effect of visual condition (EC/ EO) was highly significant (*F* = 55.6, *p* < 0.0001), whereas there were no significant interactions between (a) subjects group (PSP, IPD, CTR) and visual condition (EC/EO, *F* = 0.63, *p* = 0.53), and (b) segment, subject group, and visual condition (*F* = 0.54, *p* = 0.70, Figures 3b,c,g,h,m,n). In fact, the larger head GAIN in IPD with eyes closed (EC, Figures 3g vs. h), which applied especially for medium (0.4–0.9 Hz) and low frequencies (0.05–0.15 Hz), did not reach significance thresholds. PSP head GAIN was not different between EC and EO across the full frequency range 0.05–2.2 Hz (Figures 3m,n). PSP EO head motion was about 5x higher than CTR.

Across all subject groups (PSP, IPD, CTR), body segments (head, shoulder, hip), and visual conditions (EC/EO), there was a significant larger GAIN for small amplitudes (0.5°) with respect to larger (1°) peak-to-peak platform tilts (*F* = 224.0, *p* < 0.0001, Figures 3d,e,i,k,o,p). Moreover, this effect significantly interacted with subject groups (PSP, IPD, CTR, *F* = 45.6, *p* < 0.0001). This interaction is based on PSP patients’ largest difference of the GAIN values between 0.5° vs. 1° tilts (Figures 3o vs. p), as compared to IPD (smaller) and CTR (smallest). Platform tilt amplitudes of 0.5° and 1° in either direction further graduated the head GAIN responses between the subject groups. In IPD 0.5° low tilt amplitude was associated with higher head GAIN for lower frequencies. The GAIN peaks in IPD at 0.05–0.15 Hz and 0.4–0.7 Hz remained discernible at both tilt angles; the visual condition described above contributed predominantly to the presence of the two-pronged GAIN pattern. In PSP the 0.15–0.4 Hz head GAIN peak was most prominently expressed during the 0.5° platform tilt.

### PHASE results

PHASE lag is defined as the shift in degrees of angle between the sinus wave of the stimulus versus the response at a given frequency (phase lead of response positive degree value, phase lag negative).

**Figure 4:**
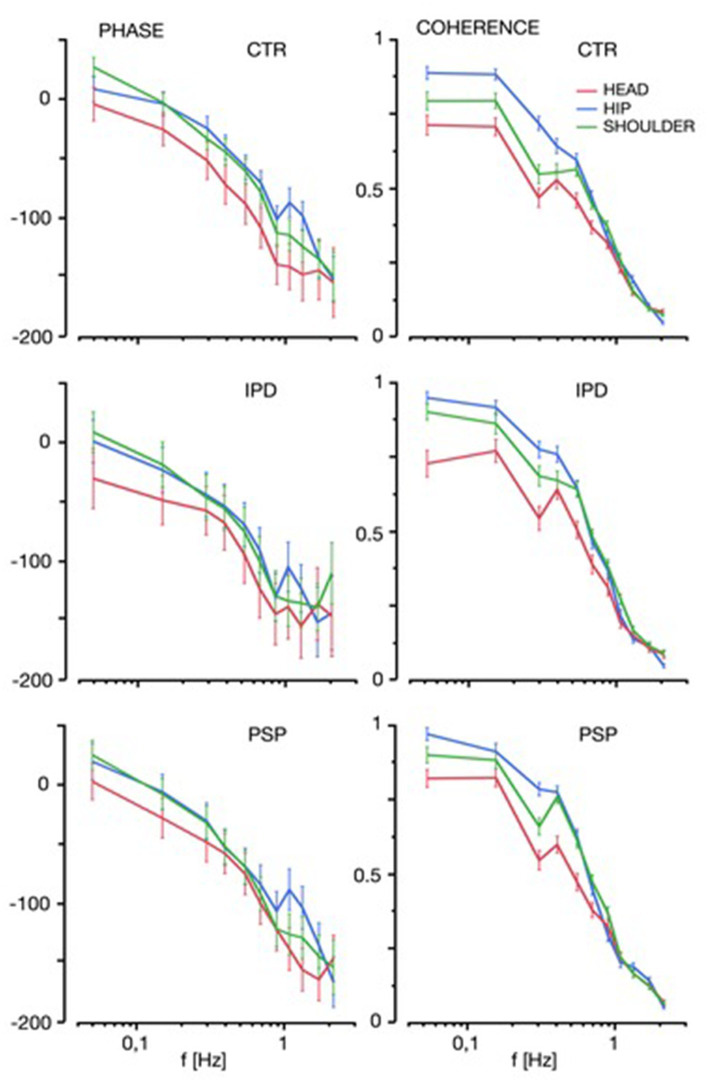
PHASE and COHERENCE of hip movements (blue), shoulder (green) and head (red) is displayed for the range of platform frequencies contained in the multi-frequency tilt stimulus among control subjects (CTR), Idiopathic Parkinson’s Disease (IPD) and Progressive Supranuclear Palsy (PSP). Vertical whiskers indicate 95% confidence intervals.

There were no significant differences between the PHASE lag between groups (*F* = 1.05, *p* = 0.35, Figure 4); PHASE lag increased at higher frequencies across all groups (effect of frequency: *F* = 77.1, *p* < 0.0001). Only at the lowest frequency of 0.05 Hz there was a small PHASE lead in all groups, which reflects known anticipatory reactions to low-amplitude low-frequency steady-state disturbance (64). Along the multi-segmental body model, phase lag of the head element was more expressed than for the lower body segments (*F* = 14.1, *p* < 0.0001).

### Coherence of GAIN and PHASE

The trial-to-trial coherence of GAIN and PHASE across the investigated frequency spectrum was a little larger in PSP and IPD, as compared to CTR (*F* = 3.76, *p* = 0.023), with consistently lower variability and higher signal to noise ratio (see Figure 4) for lower frequencies (effect of frequencies across all subject groups, segments, and visual conditions: *F* = 1304.0, *p* < 0.0001). Head trial to trial variability was statistically more expressed (through lower COHERENCE values, *F* = 117.5, *p* < 0.0001), than in the lower body segments in all groups, with no significant difference among subject groups (interaction: *F* = 2.0, *p* = 0.08). There was however a notably exaggerated variability beyond a pure linear relation for 0.3 Hz. Responses to higher frequencies were attenuated more due to head inertia across all conditions and groups (64).

**Figure 5:**
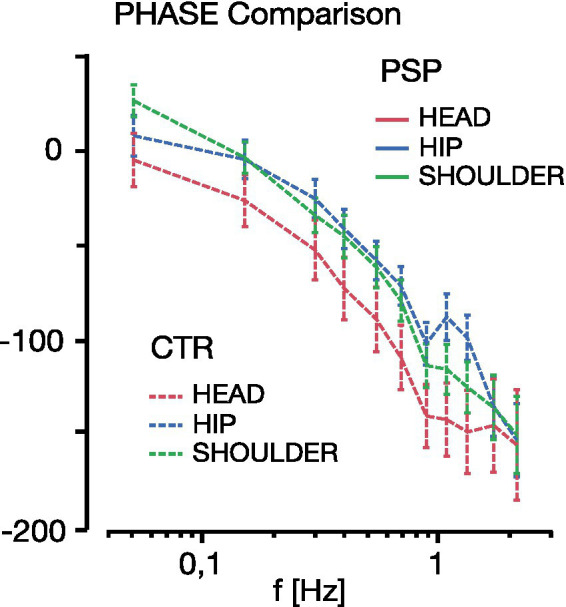
Stimulus response PHASE (in ° degree lag, negative scale) comparison between PSP (solid line) and CTR (dashed line, each with 95% whisker plots) for the body segments (head red, shoulder green, hip blue) across the platform stimulation range frequency (0.05–2.2 Hz). Note that the PHASE difference between the head (red) and shoulder (green) segments for either group does not exceed 30° lag at any frequency in either PSP or CTR. This does not support a notion of a possible 180° antiphasic head-on-shoulder motion in PSP relative to the moving platform.

The large increase of head motion GAIN in PSP exceeding both IPD two-fold and CTR by a factor of 5, and for 0.15–0.4 Hz up to a factor of 25, warranted further investigation with respect to the stimulus-dependent portion of PSP head motion. As depicted in Figure 5, PSP PHASE shift did not exceed 30° relative to the stimulus of platform motion. Particularly there was no indication that any frequency-dependent PSP head response was around 180° antiphase to platform motions, which is discussed in detail thereafter.

### Probabilistic head position

**Figure 6:**
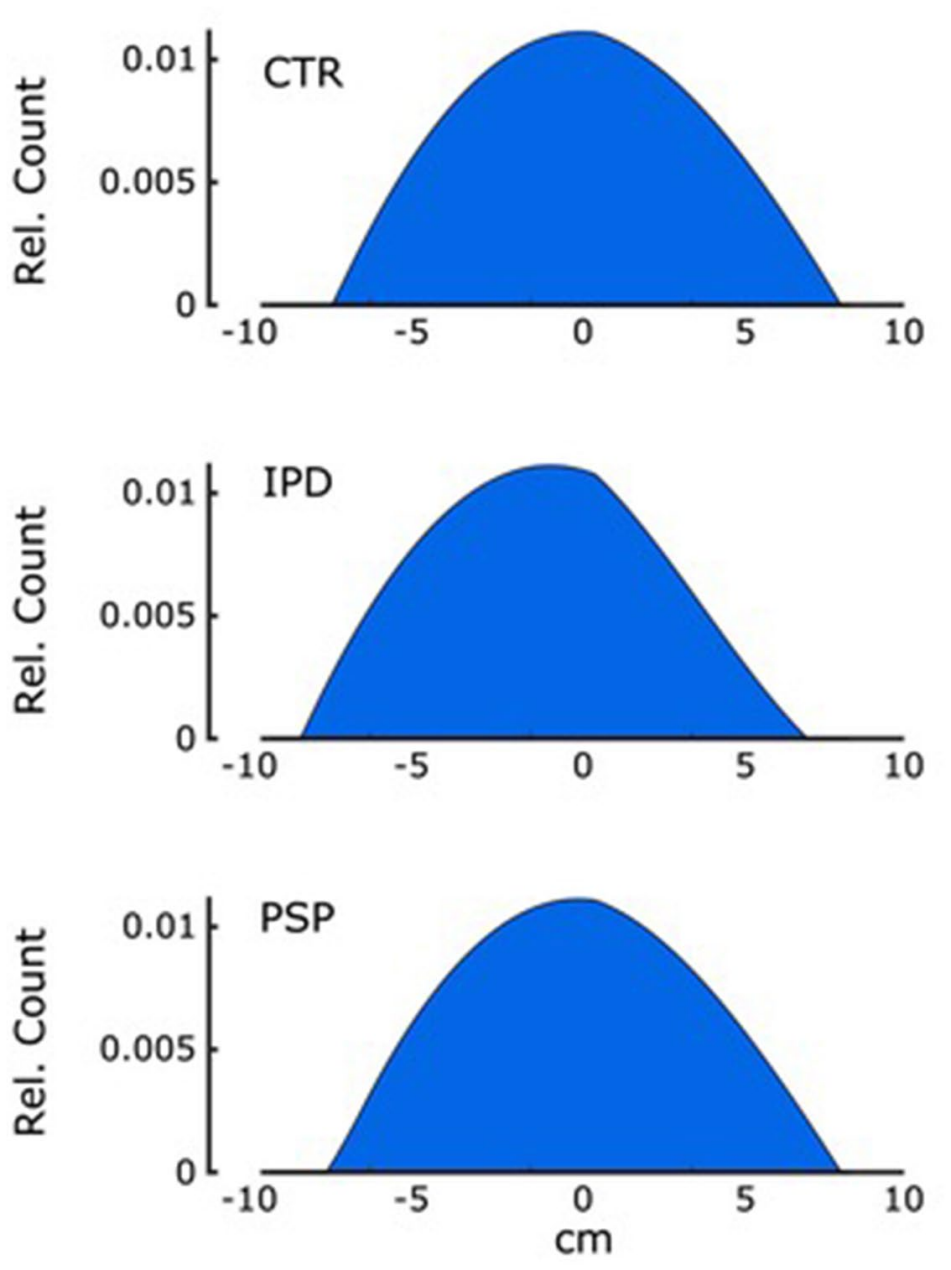
The probability distribution of the central head marker position within 10 cm each forward and backward along the anteroposterior axis of platform motion relative to its neutral position is shown with a total area under the curve of 1. Graphs are depicted for groups CTR, IPD and PSP for eyes open and closed, respectively. There was no significant difference between groups and conditions in curve parameters.

Despite relative differences in motion of the head along the neck segment and differences in the GAIN factor observed between groups, the probabilistic position of the central head marker along the axis of platform motion in conditions “eyes open “and “eyes closed” was no different between groups when assessed in 1° bins (Figure 6).

## Discussion

Present findings characterized distinct abnormalities in motor control of the head segment between clinically probable ambulatory PSP of the Richardson type, defined by the previous NINDS-PSP criteria, and late stage IPD with falling disability in relation to healthy control subjects CTR. In both patient groups, the GAIN factor of the transfer function, which describes the frequency-dependent amplification of sensory input to generate the stabilizing postural motor response of the head in space, was distinctively altered.

### Common characteristics of the head transfer function

Among all groups, head GAIN was higher than GAIN of the shoulder and hip segments in accordance with previous studies on a multi-segmental body chain [e.g., (64)]. PHASE of the imposed PRTS stimulus could be well followed by all groups. The mechanical characteristics of the body act as a low pass filter with a sharp decline beyond frequencies of 1 Hz; this can be seen from the low signal-to-noise ratio and low trial-to-trial coherence across all groups in Figure 3.

### Idiopathic Parkinson’s Disease IPD

Results for IPD underline an alignment of both body and head to a constant space alignment along the line of gravity during ground surface instability, in line with results and analyses detailed in Kammermeier et al. (56). Exclusion of visual input in the EC condition versus EO resulted in non-significantly increased GAIN of head movement in space and the overall upper body segment of head and trunk (indicated by the shoulder segment here), in accordance with findings by Vaugoyeau et al. (65). Particularly at medium (0.4–0.9 Hz) and low frequencies (0.05–0.15 Hz), movements of the platform could less be compensated for by the head. These deficits did not lead to increased relative movement in the neck segment in any condition (relative motion head versus shoulder).

### Progressive Supranuclear Palsy PSP

PSP head motion was significantly increased in relation to IPD and controls in a characteristic frequency-dependent pattern. When seen in relation to head motion in an absolute spatial reference frame, a different response pattern of head motion could be observed: a disproportionately high GAIN presented by a factor of 5-fold in comparison to CTR and up to 25-fold in the mid-frequency range 0.15–0.4 Hz, with a drop-off toward lower and higher frequencies. This increase was more pronounced in the lower maximum platform tilt amplitude of 0.5° versus 1° (Figure 3). The visual condition (eyes open EO/eyes closed EC) did not result in a particular GAIN increase during visual exclusion, however. A similar response pattern, albeit less expressed in GAIN, could be observed in IPD during the EC condition.

In our previous study (56), the overall postural response of PSP was defined as disproportionately high overall body GAIN at low <0.5 Hz and at high frequencies >1.0 Hz, higher GAIN in the combined head and trunk segment versus the lower extremities, as well as higher overall body GAIN for 0.5 versus 1° platform tilt. For overall body responses, the visual condition did not have significant effects.

The mechanisms for the significantly difference between head motion and upper body motion with respect to the platform (56) might be speculated to be caused by differences in PHASE behavior of head relative to stimulus versus trunk relative to stimulus: if the head moved synchronously and with the same amplitude as the trunk, the neck joint movement in between would be small. However, if the head moved by the same amount as the trunk, but in antiphase with respect to the earth vertical in each movement, this might have been one potential explanation of the observed large head motion. Vestibular afference of semicircular canals (acceleration sensor) allows for an up to 180° PHASE lead and otolith-derived velocity input may still amount to a 90° PHASE lead under certain conditions (64); central impairments of PSP otolith signal processing (14, 15) might amount to deficits or relevant alterations in the temporal PHASE domain. However, as detailed in the results section above, the stimulus-related portion of PSP head movements did not exceed 30° PHASE shift relative to the respective frequencies of platform motion (Figure 5). Therefore, antiphase head motion could be excluded. This notion would also contradict a specific central strategy in the stimulus-related time domain as part of a compensation or shift of sensory focus, leaving the main between-groups difference in the amplitude domain of stimulus-related GAIN.

Results of both head motion versus body and versus space indicate that PSP patients had specific alterations in controlling head segment motion in the low frequency band <0.7 Hz and particularly <0.4 Hz, which is in part low pass filtered by body mechanical properties. A particular contribution to this effect by the clinically described higher degrees of axial rigidity in PSP versus IPD was not likely in the postural context, since rigidity/stiffness would instead act mostly in the higher frequency/velocity range [see e.g., (66)]. Considering that visual-guided exploratory saccadic vertical eye movements are severely restricted and sometimes even fixed in PSP due to the mesencephalic supranuclear lesion pattern, and that vertical eye motion based on the intact infranuclear mechanism of the vestibulo-ocular reflex (VOR) arc is required to hold gaze level at the horizon during anteroposterior tilt, it should be expected that the visual condition might be a larger contributor to brainstem-centered head stabilization strategy in our task. The optic flow with respective alterations in focal depth of field conveyed through the supposedly intact VOR neurons however, by itself could not compensate for head motion versus platform stimulus, as demonstrated in Figures 3, 4. This implies a pronounced contribution of supranuclear optic field stabilization and perception among subjects with PSP for the described task.

It may be argued that optic flow with only vestibulo-ocular stabilization in the head alone with limited or no supranuclear-guided gaze targeting can no longer act as sufficiently reliable sensory input (particularly for low-frequency visual flow), and is therefore downregulated in its contribution by multisensory re-weighting toward vestibular and proprioceptive cues. This is supported by findings in our previous study (56), which suggested that PSP overall body response tended to align to a proprioceptive-based platform vertical-related coordinate frame. Scaling down the relative visual input weighting would decrease sensitivity of the overall system for low frequency and low amplitude shifts, which could in turn be more easily detected by optic flow deviation, resulting in over-compensatory responses in these conditions, as observed here. This strategy would have the side effect of exposing the head-centered semicircular canals to disproportionately increased angular accelerations, the otoliths to an increased velocity signal both as part of the vestibular system, and the neck proprioceptive sensors to larger torque angles at lower amplitudes and frequencies, giving them an amplified input in a particular frequency motion range. This might partially compensate for the loss of low frequency low amplitude visual-otolith sensor quality by repurposing a part of the sensory range of higher frequency higher amplitude sensors by means of a frequency range upshift (64).

Alternatively, these head motion characteristics could be a proprietary deficit of the disease independent from optic flow acquisition quality, since there was no significant difference in low frequency GAIN between the visual conditions. Our previous study indicated higher GAIN in the upper versus lower body segment, and therefore higher head GAIN could be a deficit graded along a somatosensory central representation in the course of the disease, affecting the head segment the most.

To investigate these characteristics more deeply, a trial with simultaneous gaze and head motion analysis in PSP would be required, particularly when presented with onscreen presentation of conflicting visual flow stimuli. Additionally, sequential assessment along the disease course would greatly aid in the description of developing deficits and possible development of temporarily effective central compensation strategies before loss of mobility.

### PSP head stabilization strategy

Given present results, two opposing theories about head motion strategy in PSP can be formed:

- Benefit theory: increased PSP head motion optimizes the measuring range of one or more sensory systems to compensate for deficits elsewhere
- Deficit theory: increased head motion is a symptom of central sensory integration deficits by central network neurodegeneration and loss of functions

Considering PHASE results of all groups with a synchronous PHASE to the stimulus and a slight PHASE lead up to 10° at 1 Hz at higher frequency components, a central active strategy is required. A solely proprioceptive-based computation does not suffice; whereas a vestibular-derived input allows up to 180° lead based on acceleration (semicircular canals) and 90° on a velocity-based signal [indirectly derived from otolith input; (64)]. However, this strategy can only function well at repetitive sinusoidal disturbance, which does not typically occur in a natural environment. Vestibular input contributes to the PSP stabilization strategy at least partially, at least by keeping PHASE in line with IPD and CTR slight PHASE lead.

PSP head motion in space is approximately double that of IPD, 5x of CTR, and is about 4x higher than a pure function of platform movements. As described previously in Kammermeier et al. (56), overall body alignment in PSP individuals approximates the moving platform vertical (egocentric reference frame), whereas individuals with IPD align to the space vertical (allocentric reference frame). Healthy control subjects (CTR) maintain a loose correlation in the middle ground between the other extremes, supposedly because healthy postural control in the investigated conditions is nowhere near challenging stability limits and may allow for less closely controlled active compensation at the low level of postural challenge. These findings by themselves favor neither benefit nor deficit theories for PSP, but only support a notion that head motion is not just a passive consequence of shoulder motion in either group, but instead requires active central strategies. To support the benefit theory, either visual and/or proprioceptive added or improved input would need to improve postural function. PSP platform vertical alignment requires: (A) active downregulation of vestibular inputs in alignment with the benefit theory or (B) deficit or lack of vestibular signal integration (deficit theory).

Visual input through the EO/EC condition testing was indicated to be an additional limited aid to IPD for the entire upper body segment (56), and only at near-significant levels for the head segment in this study. This was of no relevance for PSP, neither in the absolute spatial or neck joint relative reference frame. A small proportion of visual input still aided PSP within their egocentric referencing, as measured in GAIN/PHASE relations, but nowhere near functionally adequate compensation. Physiologically, this may relate to midbrain degeneration of supracollicular tracks related to optic flow processing. Loss of supranuclear-guided vertical eye motility by degeneration of the responsible vertical eye movement center riMLF (Nucleus of the rostral interstitial mediolateral fascicle) likely contributes additionally; the exact interaction of head and eye motion in these conditions however will require additional experimentation, particularly by simultaneous recording of eye movements during dynamic posturography. Studies with fMRI in association with gait tasks revealed decreased metabolism in the thalamic and oculomotor-related midbrain tegmental metabolism among PSP with higher falling disposition (67–69), associating decreased supranuclear oculomotor capabilities with worsened postural performance.

Proprioceptive input at the neck level was shown to be sufficiently functional in the same group of patients investigated here by experimental neck vibration (57). Considering proprioceptive input along the whole body are referenced centrally (54), it may be suggested that slightly higher angles (1° tilt vs. 0.5° tilt) within stability limits would result in more robust signal-to-noise ratio of the joint and muscle sensors, and therefore improved body stabilization. However, no differences in the quality or amount of postural compensation could be observed in the PSP group for the 1° vs. 0.5° condition alone, or paired with the variables 1°/0.5°*EO/EC.

The exceedingly scaled head motion in PSP patients on top of surface-centered body alignment was also not directed at keeping the head stabilized in relation to space or a certain motion range of the neck, which would indicate an optimization strategy. Additional pointers toward a benefit theory may hail from the notion of clinically relevant postural performance improvements in PSP with vestibular stochastic resonance therapy [noisy galvanic vestibular stimulation nGVS, (70)], which supposedly can improve vestibular acuity. Improvements of vestibular acuity on the scale observed by Wuehr and colleagues seem plausible to improve central compensation considerably when the respective sensory quality is already target of an underlying optimization strategy in the amplitude domain (GAIN). Signal contrast enhancements to a defectively acting input appear less likely to have such large effects.

Further studies aimed at the time course of PSP-RS and other subtypes of PSP are required to differentiate between benefitting compensation strategies, progress of central deficits and their relative prevalence over time.

Limitations of this study include the original clinical classification of the PSPRS used at inclusion of PSP patients tailored toward the Richardson subtype PSP-RS, which forms the most frequent and typical clinical syndrome, omitting data on, e.g., PSP-P (13, 22) and less frequent variants with their respective motion control deficits. This was due to the collective of patients in this particular study enlisted from the concomitant PROSPERA study. The clinical classification has by now been updated to Movement Disorders Society PSP criteria (13), also featuring classification of PSP subtypes and a foundation to include them in studies like these. The measurements described here were taken at individual time points during disease stages leading up to eventual immobilization. Obtaining several recording instances over a period of months among the same individuals may further elucidate more specific determinants of loss of postural stability. PSP subtypes such as PSP-P with their initial Levodopa response and further subtypes should be included in a broader scope of the disease, possibly differentiating specific central strategies or failures. Finally, the mode of data acquisition by 3D ultrasound markers provided a temporal and spatial resolution outdated by present 3D camera motion capture systems augmented by strategic placement of accelerometers and video-oculography systems, which would allow to further investigate the role of supranuclear and brainstem eye motion in overall stance motor control. Future studies should aim to complete the picture on this particular scope of Parkinsonism postural control. Findings may implicate refined understanding of resilience of upright stance in neurological diseases and possibly robotic motor control and sensory integration. Computerized algorithms (neural networks and/or support vector machines) trained to the different postural control characteristics and their alterations in the disease course may eventually aid in the differential diagnosis during early stages of the respective diseases (e.g., clinical onset PSP-P versus early IPD).

## Conclusion

Overall, the focus of PSP patients on egocentric stabilization leads to a loss of degrees of freedom in postural stabilization and a deficit in the central integration particularly of vestibular more than visual inputs. Head movements are disproportionately increased, but fail to improve the measuring range and effect of the head-based vestibular and visual systems. PSP respond to miniscule floor perturbation with exaggerated head motion in the anteroposterior plane phased against the movements of the trunk. This active strategy however does not appear to contribute to improved sensory characteristics of postural control mechanisms.

Pathoanatomically, the heaviest burden of PSP neurodegeneration lies in the midbrain area, a site of multisensory integration with supracollicular-mediated optic flow, ascending vestibular information in the mediolateral fascicle MLF, vertical gaze control in the rostral interstitial nucleus riMLF and descending spinal control pathways, including the rubrospinal and parts of the reticulospinal tracts. The integration of proprioceptive input through the cerebellum, parts of vestibular afference and the descending vestibulospinal tract are largely spared at least in early PSP (2). Results presented here indicate that the integration of visual and vestibular processing into proprioceptive information appears to be severely lacking already in early PSP, whereas proprioceptive and basic vestibular PHASE lead characteristics appear to be largely spared.

### Significance

Evidence of abnormally large head excursions in PSP-RS patients during the postural context contradicts the concept of increased axial rigidity as a major culprit for falling in the early disease stage, despite PSP patients expressing typically pronounced neck rigidity in comparison to IPD. These head excursions likely reflect degeneration of visual flow processing (71) and integration of vestibular information; the remaining dysfunctional strategy aligns the body with the vertical of an unstable surface, mainly based on proprioceptive sensory input. Head motion is particularly large in amplitude, without direct indications of sensory acquisition or processing benefits to other sensory afferents to aid overall postural stability. These insights may aid in developing compensatory therapies by specialized physiotherapy and defines measurable outcome parameters for future experimental therapies.

Both IPD and PSP display disproportionally high head GAIN across all testing conditions for frequencies <0.7 Hz with high variability. Particularly PSP exhibits prominent GAIN amplifications in the 0.15–0.4 Hz range.

IPD reacts in both “eyes open” (EO) and “eyes closed” (EC) conditions with a disproportional increase of head GAIN of medium (0.4–0.9 Hz) and low frequencies (0.05–0.15 Hz) in EC (Figure 3h), which amounts largely to the overall GAIN response of combined conditions in Figure 3f, whereas GAIN response of IPD during EO mimics the frequency of CTR with higher GAIN and wider distribution. In IPD, amplitude 0.5° is associated with higher head GAIN in an inverse relationship with frequency (Figure 3i). The GAIN peaks in IPD at 0.05–0.15 Hz and 0.4–0.7 Hz remain prominent at both tilt angles (Figures 3i–k).

PSP show their exaggerated GAIN response irrespective of the visual condition (EO versus EC, Figures 3m,n) with no significant difference at any frequency (Figures 3o,p). In PSP the 0.15–0.4 Hz head GAIN peak is prominently due to the 0.5° platform tilt.

Please note that the presented data represents the output of the central multisensory transfer function instead of absolute head motion relative to body or absolute space.

Group PHASE responses reflect combined conditions of “eyes open” (EO) and “eyes closed” (EC) during 0.5° and 1° maximal angle platform tilts pooled. There are no significant differences between the PHASE lag between groups; PHASE lag increases at higher frequencies and only at the lowest frequency of 0.05 Hz there was a small anticipatory PHASE lead.

A COHERENCE value of “1” indicates completely identical responses of the respective body segment in each iteration and optimal signal to noise ratio, whereas values approaching “0” correspond to highly variable trial-to-trial responses and low signal to noise ratio.

As in Figure 3, please note that data represents the output of the central multisensory transfer function instead of absolute head motion relative to body or absolute space.

## Data Availability

The raw data supporting the conclusions of this article will be made available by the authors, without undue reservation.

## Footnotes

^1^ www.matlab.com

^2^ www.zebris.de

## Supplementary material

SUPPLEMENTARY 1 Transfer functions (numbered 2–7 in the original publication) describing the stimulus-derived motion of the multisegmental inverted pendulum model, as laid out by Peterka (64).
