# Supplementary figures and images for: Head position control strategies in progressive Supranuclear Palsy versus Idiopathic Parkinson’s Disease during dynamic-on-static platform tilt
Source: Front Neurol. 2025 Apr 16;15:1477493. doi: 10.3389/fneur.2024.1477493 (PMC12040646; doi:10.3389/fneur.2024.1477493)

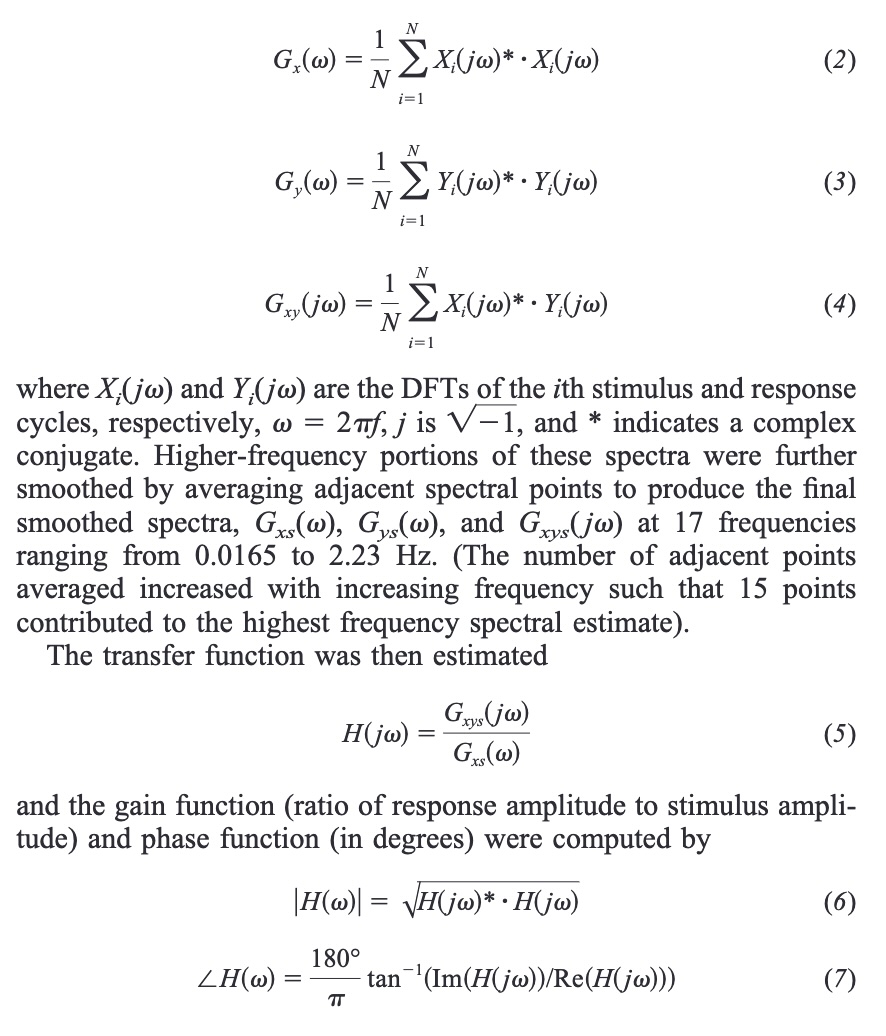

Supplement: SUPPLEMENTARY 1 — Transfer functions (numbered 2–7 in the original publication) describing the stimulus-derived motion of the multisegmental inverted pendulum model, as laid out by Peterka (64). [file Image_1.JPEG]
